# Effects of Simvastatin on Glucose Metabolism in Mouse MIN6 Cells

**DOI:** 10.1155/2014/376570

**Published:** 2014-06-04

**Authors:** Jieqiong Zhou, Weihua Li, Qiang Xie, Yuxi Hou, Shaopeng Zhan, Xi Yang, Xiaofeng Xu, Jun Cai, Zhengrong Huang

**Affiliations:** ^1^Department of Cardiology, The First Affiliated Hospital of Xiamen University, 55 Zhenhai Road, Xiamen 361003, China; ^2^Department of Cardiology, Chaoyang Hospital, Capital Medical University, 8th Gongtinanlu Road, Chaoyang District, Beijing 100020, China

## Abstract

The aim of this study was to investigate the effects of simvastatin on insulin secretion in mouse MIN6 cells and the possible mechanism. MIN6 cells were, respectively, treated with 0 **μ**M, 2 **μ**M, 5 **μ**M, and 10 **μ**M simvastatin for 48 h. Radio immunoassay was performed to measure the effect of simvastatin on insulin secretion in MIN6 cells. Luciferase method was used to examine the content of ATP in MIN6 cells. Real-time PCR and western blotting were performed to measure the mRNA and protein levels of inward rectifier potassium channel 6.2 (Kir6.2), voltage-dependent calcium channel 1.2 (Ca_v_1.2), and glucose transporter-2 (GLUT2), respectively. ATP-sensitive potassium current and L-type calcium current were recorded by whole-cell patch-clamp technique. The results showed that high concentrations of simvastatin (5 **μ**M and 10 **μ**M) significantly reduced the synthesis and secretion of insulin compared to control groups in MIN6 cells (*P* < 0.05). ATP content in simvastatin-treated cells was lower than in control cells (*P* < 0.05). Compared with control group, the mRNA and protein expression of Kir6.2 increased with treatment of simvastatin (*P* < 0.05), and mRNA and protein expression of Ca_v_1.2 and GLUT2 decreased in response to simvastatin (*P* < 0.05). Moreover, simvastatin increased the ATP-sensitive potassium current and reduced the L-type calcium current. These results suggest that simvastatin inhibits the synthesis and secretion of insulin through a reduction in saccharometabolism in MIN6 cells.

## 1. Introduction

Statins are inhibitors of 3-hydroxy-3-methyl-glutaryl coenzyme A (HMG-CoA) reductase that can reduce the plasma level of total cholesterol, low density lipoprotein cholesterol, and glycerin trilaurate and increase high density lipoprotein cholesterol [1]. In the past two decades, more than 170,000 participants in a number of large-scale clinical trials demonstrated that statins reduce the incidence of cardiovascular events [2]; thus, statins are now widely used for the primary and secondary prevention of both atherosclerotic cardiovascular disease (ASCVD) and stroke [3–6]. However, in contrast to its clear benefit to cardiovascular disease prevention, a substantial number of meta-analyses of large, randomized controlled clinical trials showed that statins also increased the risk of new onset diabetes, in comparison to the placebo group [7–9]. The mechanism by which statins cause this increase in type 2 diabetes risk is unknown. Accordingly, in this study, we investigated the effects of statins on the two known mechanisms that cause type 2 diabetes: insulin resistance and the dysfunction of islet beta cells. Thus, we tested the effects of simvastatin on insulin secretion in the mouse islet beta cell line, MIN6, and explored the mechanism of this function.

## 2. Materials and Methods

### 2.1. Reagents

The mouse islet beta cell line, MIN6, was a gift from the Ruijin Hospital, affiliated to Shanghai Jiao Tong University School of Medicine, Institute of Endocrinology. Simvastatin was obtained from MERCK; antibodies against inward rectifier potassium channel 6.2 (Kir6.2) and GLUT2, as well as the HRP-conjugated rabbit polyclonal antibody, were purchased from Abcam. The antibody against Ca_v_1.2 was obtained from Novus; the antibody against glyceraldehyde-3-phosphate dehydrogenase (GAPDH) was obtained from Cell Signaling. The ATP detection assay kit was obtained from the Beyotime Institute of Biotechnology.

The formulation of the extracellular fluid for the K_ATP_ channel currents (*I*_KATP_) was as follows: 80 mM NaCl; 60 mM KCl; 10 mM HEPES; 1 mM MgCl_2_; 0.1 mM CaCl_2_ (pH 7.4, NaOH). The electrode internal fluid for these K_ATP_ channel currents contained the following: 15 mM NaCl; 92 mM KCl; 33 mM KOH; 10 mM HEPES; 1 mM MgCl_2_; 1 mM CaCl_2_; 10 mM EGTA; 0.2 mM MgATP (pH 7.2, KOH) [1].

The formulation of the extracellular fluid for the L-Ca channel currents (*I*_Ca-L_) was as follows: 30 mM BaCl_2_; 99.3 mM CsCl; 16.7 mM glucose; 10 mM tetraethylammonium chloride (TEACl); 1.2 mM MgCl_2_; 10 mM Hepes (pH 7.4, CsOH). The electrode internal fluid for these L-Ca channel currents contained the following: 130 mM CsCl; 10 mM TEACl; 5 mM MgCl_2_; 4 mM ATP-Na_2_; 0.4 mM GTP-Na_2_; 10 mM EGTA; 10 mM Hepes (pH 7.2, CsOH) [2].

All solutions were filtered through a microporous membrane (0.22 *μ*m).

### 2.2. Cell Culture

MIN6 cells were cultured in DMEM supplemented with 15% FBS, 100 U/mL penicillin, 100 *μ*g/mL streptomycin, 2 mM L-glutamine, and 70 *μ*mol/L beta-mercaptoethanol. Cells were divided into NC, A, B, and C groups at 6 × 10^5^/mL; medium was changed every two days, and A, B, and C groups were treated with 2, 5, and 10 *μ*M simvastatin, respectively, for 48 h. NC was the normal control group.

### 2.3. Insulin-Releasing Test and Detection of Insulin Content in MIN6 Cells

Cells were cultured in 12 wells at 5 × 10^4^/mL for 48 h and then washed with KRBB (115 mM NaCl, 4.7 mM KCl, 1.2 mM MgSO_4_, 1.2 mM KH_2_PO_4_, 1.3 mM CaCl_2_, 24 mM NaHCO_3_, 0.1% BSA, and 10 mM Hepes, pH 7.4) twice; glucose-free Krebs-Ringer buffer was added for 2 h at 37°C, and then the medium was removed. Krebs-Ringer buffer with 2.8 mM and 16.7 mM was added into each group for 1 h at 37°C; then the supernatant was removed, and the cells were centrifuged at 1000 rpm for 5 min at 4°C; then the supernatant was used for immediate testing or stored at −70°C. Equal acid alcohol (75% ethanol, 1.5% hydrochloric acid, and 75% ultrapure water) was added to extract the cells, and then they were stored at 4°C. In the following day, insulin was measured in the supernatant, according to the kit, and the results were adjusted with total protein concentration. Each experiment was repeated three times.

### 2.4. Measurement of ATP Content in MIN6 Cells

Cells were cultured in 12 wells; 100 *μ*L cell lysis buffer was added to each well, and then the lysed cells were centrifuged at 12,000 g for 8 min. The supernatant was collected; 100 *μ*L ATP working solution was added to the tube at room temperature for 5 min, and then 20 *μ*L sample buffer was added and mixed quickly; then the RLU was read with a luminometer.

### 2.5. Real-Time PCR

Total RNA was isolated using the Trizol reagent, according to the manufacturer's instructions. The cDNA was synthesized from 2 *μ*g of total RNA, using MMLV transcriptase with random primers. Real-time PCR was performed using SYBR Premix ExTaq. Quantification was normalized to the amount of endogenous GAPDH. Primers used for real-time PCR are listed in Table 1.

### 2.6. Western-Blot Analysis

Cells were lysed with lysis buffer (200 mM Tris-HCl (pH 7.5), 1.5 M NaCl, 10 mM EDTA, 25 mM sodium pyrophosphate, 10 mM glycerol phosphate, 10 mM sodium orthovanadate, 50 mM NaF, and 1 mM PMSF, in combination with a protein inhibitor cocktail). 30 *μ*g of protein lysates from each sample was subjected to SDS-PAGE and transferred onto nitrocellulose membranes. Blots were incubated with the specific primary antibodies overnight at 4°C. After washing them three times for 15 min each with TBST (TBS + 0.1% Tween20), blots were incubated with a horseradish peroxidase-conjugated secondary antibody (Pierce, Rockford, IL, USA) and visualized by chemiluminescence. Band densities were quantified by densitometry using the Scion Image software and normalized to *β*-actin levels.

### 2.7. *I*_KATP_ and* I*_Ca-L_ Recordings Using the Whole-Cell Patch-Clamp Technique

A coverslip containing adherent cells was mounted in a recording chamber on an Olympus IX 51 microscope (Olympus, Tokyo, Japan). Glass capillaries (Sutter, USA) were used to fabricate patch electrodes through a micropipette puller (P97, Sutter, USA), which had resistances of 2 to 5 MΩ, when filled with an intracellular solution. The cells were washed with extracellular solution for 2 min, and the patch electrodes, which were attached to the MIN6 cell (well-grown, smooth-faced, and tile-shaped), were moved using a motorized micromanipulator (MP-225, Sutter, USA). First, the liquid junction potential was eliminated; then the electrode capacitance transients were compensated, and, after gigaseals (>1 GΩ), fast capacitance was established by negative pressure. Using further suction to break the membrane, a whole-cell recording was formed. Finally, slow capacitance was compensated. Membrane capacitance around 7~9 pF was selected as the last record.

The* I*_KATP_ was elicited from a holding potential of −70 mV, with a prepulse to −40 mV for 500 ms, and with test potentials ranging from −120 mV to +80 mV in 20 mV increments during 3000 ms. The *I*_Ca-L_ was elicited from a holding potential of −70 mV, a prepulse to −70 mv for 500 ms, and depolarized from −40 to +50 mV in 10 mV increments during 3000 ms. Calcium currents were recorded within 5–20 minutes.

All recordings were performed at room temperature (~23°C), with a 100 *μ*s sampling interval, and digitally filtered at 1 kHz. Leak subtraction for gating currents was performed using the P/n protocol, as implemented in patch machine. All patch-clamp data were acquired with a patch-clamp amplifier (AXON 700B, USA), digitized with a Digidata 1440A analog-digital converter (Molecular Devices, USA), and analyzed with pClamp9+clampfit software.

### 2.8. Statistical Analysis

Data were collected from several independent experiments, with three replicates per experiment. Quantitative data are expressed as mean ± standard deviation (SD). Data were analyzed using one-way ANOVA with Tukey's posttest or nonparametric tests in SPSS 15.0. *P* < 0.05 was considered statistically significant. Bars in the graph represent SD.

## 3. Results

### 3.1. The Effects of Simvastatin on Insulin Content and Secretion in MIN6 Cells

**Figure 1:**
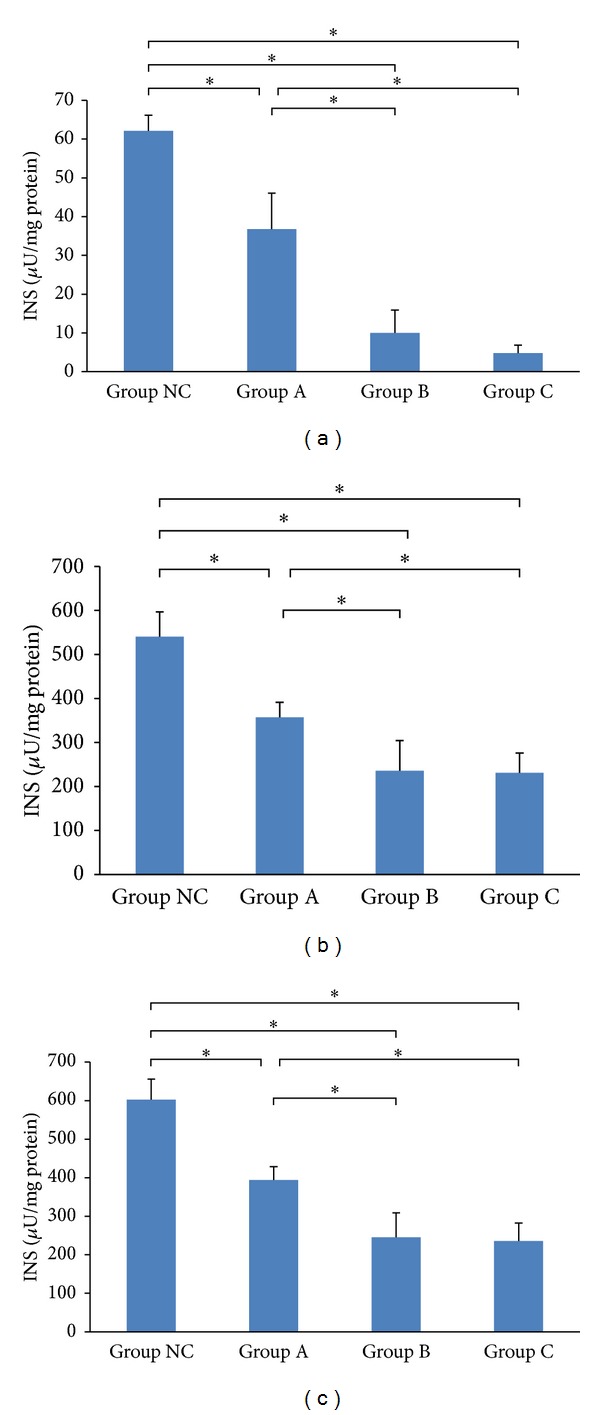
Effects of simvastatin on insulin content at low glucose levels. (a) Insulin secretion. (b) Insulin content in MIN6 cells. (c) Total amount of insulin. INS indicates insulin-releasing test. *P < 0.05.

Comparing with the normal control cells, the cells treated with simvastatin had less insulin content, less insulin secretion, and less total amount of insulin induced from a low level of glucose (*P* < 0.05). Furthermore, simvastatin decreased insulin secretion in a concentration-dependent manner (Table 2 and Figure 1).

**Figure 2:**
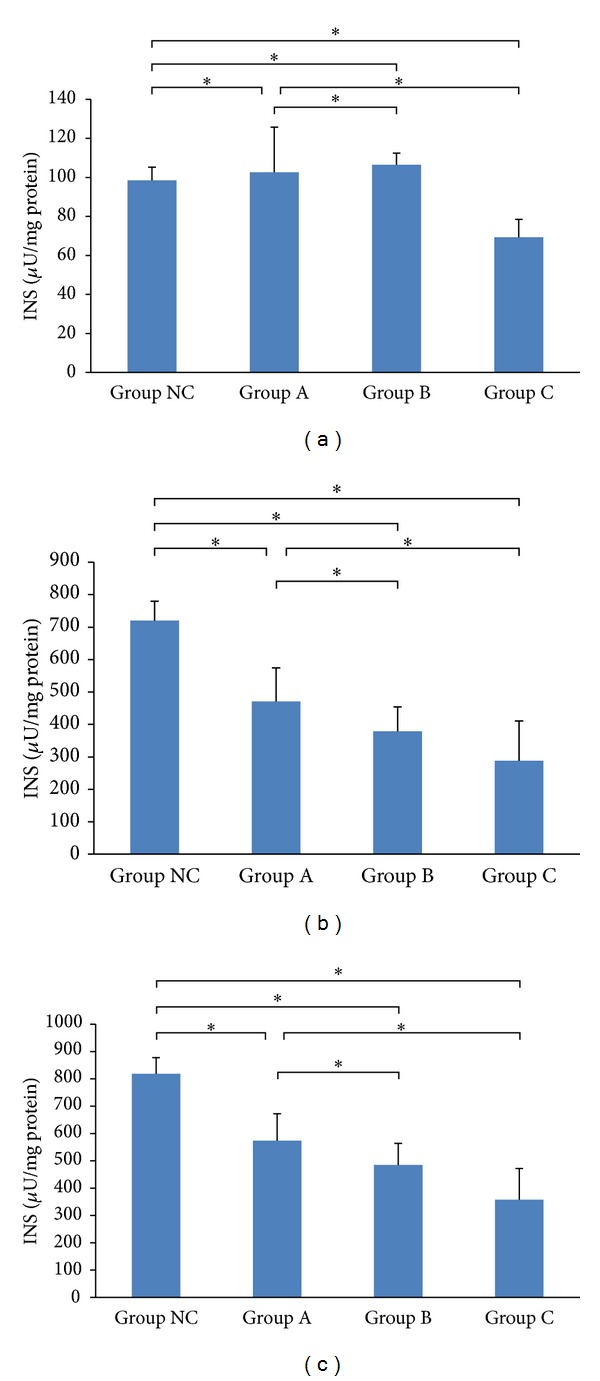
Effects of simvastatin on insulin content at high glucose level. (a) Insulin secretion. (b) Insulin content in MIN6 cells. (c) Total amount of insulin. INS indicates insulin-releasing test. *P < 0.05 and **P < 0.01.

Comparing with the normal MIN6 cells, cells treated with simvastatin had less insulin content, less insulin secretion, and less total amount of insulin induced at high glucose levels (*P* < 0.05). Furthermore, simvastatin reduced the total amount of insulin in a concentration-dependent manner. The group treated with 10 *μ*M simvastatin had a greater decrease in cellular insulin content, insulin secretion, and total insulin, in comparison to the group treated with 2 *μ*M simvastatin under high glucose levels (*P* < 0.05, Table 3 and Figure 2).

### 3.2. The Effects of Simvastatin on ATP Levels in MIN6 Cells

**Figure 3:**
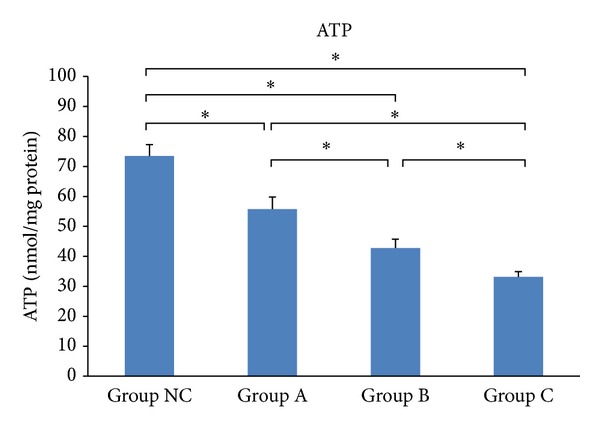
Effects of simvastatin on ATP levels in MIN6 cells. *P < 0.05.

ATP levels decreased in MIN6 cell lysates after simvastatin treatment, in comparison to the normal control cell lysates (*P* < 0.05). Furthermore, simvastatin caused this decrease in ATP in a concentration-dependent manner (*P* < 0.05, Table 4 and Figure 3).

### 3.3. The mRNA Levels of Kir6.2, Ca_v_1.2, and GLUT2

**Figure 4:**
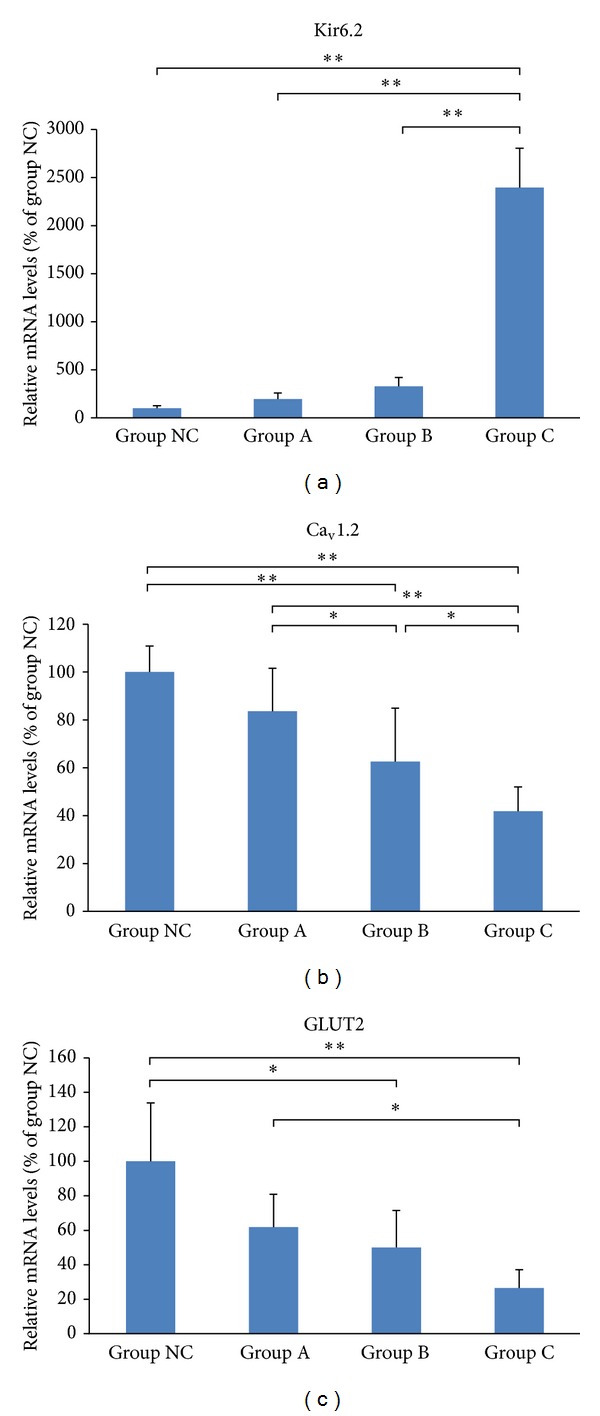
Effects of simvastatin on mRNA levels of Kir6.2 (a), Ca_v_1.2 (b), and GLUT2 (c) in MIN6 cells. *P < 0.05  and  **P < 0.01.

Compared with the normal control cells, the mRNA levels of Kir6.2 increased (23.97 ± 4.09, *P* < 0.001) in cells treated with 10 *μ*M simvastatin, while the mRNA levels of Ca_v_1.2 and GLUT2 decreased (0.63 ± 0.22, *P* = 0.002 and 0.50 ± 0.21, *P* = 0.023, resp.) in cells treated with 5 *μ*M simvastatin and were further reduced in cells treated with 10 *μ*M simvastatin (0.42 ± 0.10, *P* < 0.001 and 0.26 ± 0.11, *P* < 0.001, resp.). Kir6.2 mRNA levels were significantly higher in the 10 *μ*M treatment group than in the 2 *μ*M and 5 *μ*M groups (*P* < 0.01). The Ca_v_1.2 mRNA levels were significantly lower in the 10 *μ*M treatment group than in the 2 *μ*M and 5 *μ*M groups (*P* < 0.001, *P* = 0.038). Furthermore, Ca_v_ 1.2 mRNA levels were more significantly downregulated in the 5 *μ*M group than in the 2 *μ*M group (*P* = 0.036). GLUT2 mRNA levels were more significantly reduced in the 10 *μ*M group than in the 2 *μ*M group (*P* = 0.013, Figure 4).

### 3.4. Protein Expression of Kir6.2, Ca_v_1.2, and GLUT2

**Figure 5:**
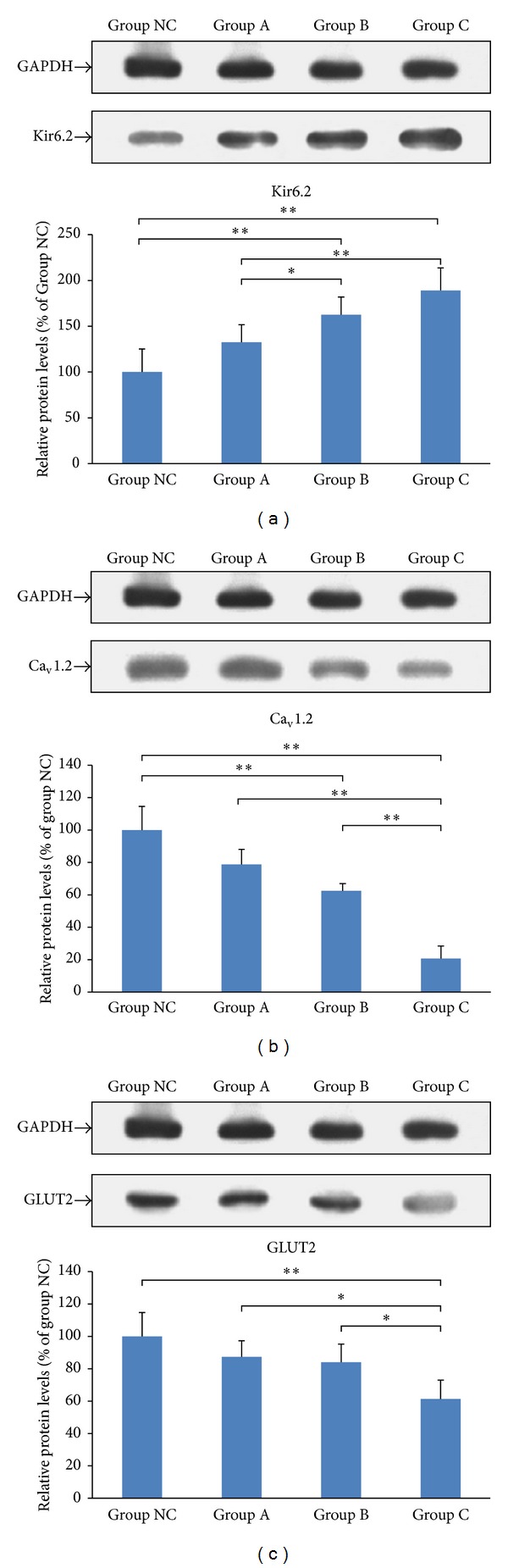
Effects of simvastatin on protein levels of Kir6.2 (a), Ca_v_1.2 (b), and GLUT2 (c) in MIN6 cells. *P < 0.05  and **P < 0.01.

As shown in Figure 5, 5 *μ*M and 10 *μ*M simvastatin increased the protein expression of Kir6.2, while the expression of Ca_v_1.2 decreased, in comparison to the control MIN6 cells. The protein expression of GLUT2 decreased in cells treated with 10 *μ*M simvastatin, in comparison to the control MIN6 cells.

### 3.5. The Effect of Simvastatin on* I*_KATP_ in MIN6 Cells

**Figure 6:**
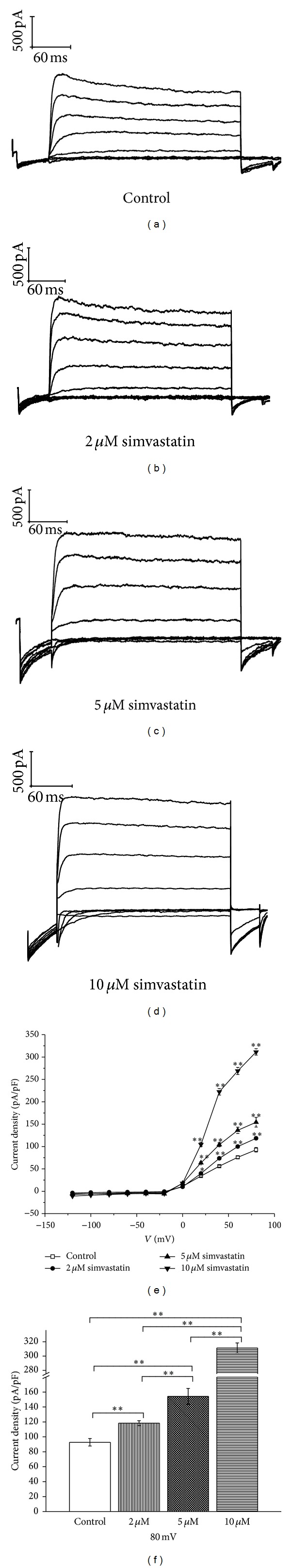
Effects of simvastatin on* I*_KATP_ and current density in MIN6 cells (*P < 0.05, **P = 0.03, and ***P < 0.01). (a) KATP current of control group (*n* = 12), (b) KATP current with application of 2 *μ*mol/L simvastatin (*n* = 12), (c) KATP current with application of 5 *μ*mol/L simvastatin (*n* = 10), (d) KATP current with application of 10 *μ*mol/L simvastatin (*n* = 8), (e) current density-voltage (*J*-*V*) curves of four groups, and (f) the current densities of cells with application of different concentrations of simvastatin at 80 mV voltage.

Simvastatin increased* I*_KATP_ in a concentration-dependent manner. At 80 mV electric voltage,* I*_KATP_ increased by 118.36 ± 3.30 pA/pF, 154.16 ± 10.72 pA/pF, and 311.37 ± 7.06 pA/pF at 2 *μ*M, 5 *μ*M, and 10 *μ*M simvastatin, respectively (Figure 6).

### 3.6. The Effect of Simvastatin on* I*_Ca-L_ in MIN6 Cells

**Figure 7:**
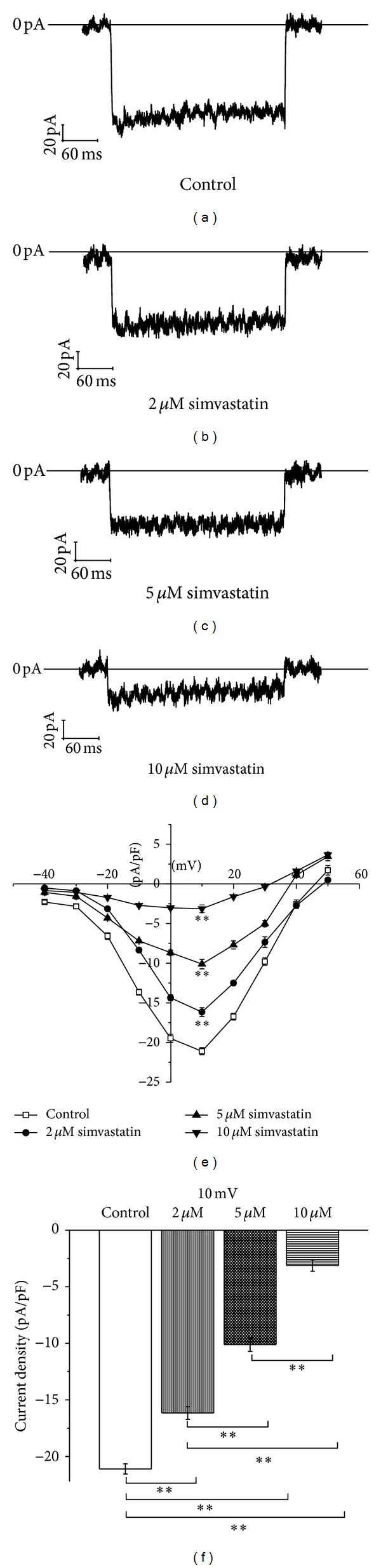
Effects of simvastatin on *I*_Ca-L_ and current density in MIN6 cells. (a) *I*_Ca-L_ current of control group cells at 10 mV, (b) *I*_Ca-L_ current of cells with application of 2 *μ*mol/L simvastatin at 10 mV, (c) *I*_Ca-L_ current of cells with application of 5 *μ*mol/L simvastatin at 10 mV, (d) *I*_Ca-L_ current of cells with application of 10 *μ*mol/L simvastatin at 10 mV, (e) current density-voltage (*J*-*V*) curves of four groups, and (f) the *I*_Ca-L_ current densities of cells with application of different concentrations of simvastatin at 10 mV voltage.

As shown in Figure 7, at 10 mV, simvastatin significantly decreased the *I*_Ca-L_ current density in a concentration-dependent manner. The current densities were −16.16 ± 0.56 pA/pF, −10.11 ± 0.59 pA/pF, and −4.71 ± 0.19 pA/pF in MIN6 cells treated with 2 *μ*M, 5 *μ*M, and 10 *μ*M simvastatin.

## 4. Discussion

Statins inhibit cholesterol synthesis, reduce plasma cholesterol levels to stabilize the atherosclerotic plaque in blood vessels, and inhibit the activity of HMG-CoA reductase in the cholesterol synthesis process. Statins are widely used in a great number of medical fields, including cardiology, neurology, nephrology, and endocrinology. Recently, there has been a great deal of attention on the effect of statins, especially in enhancer dosages, on glycometabolism, but there have been few studies on the mechanism of how statins increase diabetes risk.

The mechanism of statins on glycometabolism, decreased insulin sensitivity, and diabetes risk increase is still unknown. Nakata et al. [10] found that atorvastatin reduces insulin sensitivity by inhibiting adipose cell maturation and the expression of glucose transporter-4. The side effect of statins on glycometabolism may be related to a decrease in metabolite production. Coenzyme Q10 plays a critical role in energy metabolism; it not only is important for antioxidation and cell membrane stabilization, but also participates in mitochondrial oxidative phosphorylation [11]. Some statins have been shown to significantly reduce plasma coenzyme Q10 level, leading to a reduction in mitochondrial oxidative phosphorylation and decreased ATP production [12–15]. Furthermore, statins can inhibit the mevalonate pathway, thus preventing the production of mevalonate metabolites, such as isoprenoid and coenzyme Q10, and affecting glycometabolism and insulin sensitivity.

Insulin synthesis and secretion are a complete and orderly process in pancreatic islet beta cells. At present, the consensus on glucose-stimulated insulin secretion is that beta cells are electrically excitable; GLUT2 transports glucose into the cells, and then glucokinase phosphorylates it to ATP. These events result in ATP-sensitive potassium channel (K_ATP_) closure and membrane depolarization, voltage-dependent calcium channel (Ca_v_) opening, calcium influx, intracellular calcium concentration increase, and activation of a series of enzymes and proteins, which are necessary for insulin secretion. Therefore, many factors and stages affect insulin secretion in beta cells. Our study shows that, after 48 h of treatment, simvastatin significantly inhibited glucose-stimulated insulin synthesis and secretion at basal (2.8 mM) and high glucose levels (16.7 mM), in a dose-dependent manner in MIN6 cells.

In this study, we investigated the mechanism of how simvastatin inhibits insulin secretion in islet beta cells. ATP is a very important regulator of insulin secretion in islet beta cells; reduction of ATP inhibits the K_ATP_ channel closure, membrane depolarization, and subsequent Ca_v_ channel opening, thus inhibiting insulin secretion. Our results demonstrated that simvastatin significantly inhibited ATP production in a dose-dependent manner in islet beta cells.

K_ATP_ is an octamer, which consists of four Kir6.2 subunits and four thiourea receptor-1 (SUR1) subunits. K_ATP_ plays a critical role in the insulin secretion process by coupling glucose metabolism and electrical activity in beta cells [16]. The effects of K_ATP_ are dependent on the expression of Kir6.2 and SUR1 [17]. In addition, Ca_v_, in its relation to the L-type non-L-type calcium channels, plays an important role in insulin secretion.

Our study showed that simvastatin significantly increased Kir6.2 expression, while it decreased Ca_v_1.2 and GLUT2 expression at 5 *μ*M and 10 *μ*M treatment for 48 h. The patch-clamp technique results showed that simvastatin increased the K_ATP_ current, while it reduced the L-Ca current in MIN6 cells. Kir6.2 is an inward rectifier potassium channel, which plays important roles in membrane resting potential maintenance and insulin secretion in beta cells. Thus, by increasing Kir6.2, while decreasing Ca_v_1.2, and subsequently inhibiting depolarization and calcium influx, simvastatin is able to reduce insulin secretion. Furthermore, simvastatin can also indirectly inhibit insulin secretion by decreasing GLUT2 expression, resulting in reduced glucose uptake.

Collectively, although it is clear that statins have beneficial effects on cardiovascular disease prevention, our study supports the evidence that long-term use of large doses of statins has adverse effects on glucose metabolism and insulin sensitivity. Therefore, patient blood glucose levels should be monitored to reduce these adverse events caused by statins.

## 5. Conclusions

This study has notable strengths. We found that simvastatin inhibits the synthesis and secretion of insulin through a reduction in saccharometabolism in beta cells.

## Figures and Tables

**Table 1:** Real-time PCR primers.

Gene	Forward primers	Reverse primers
Kir6.2	AAGGGCATTATCCCTGAGGAA	TTGCCTTTCTTGGACACGAAG
Ca_v_1.2	AGACGCTATGGGCTATGA	AACACCGAGAACCAGATTTA
GLUT2	TCAGAAGACAAGATCACCGGA	GCTGGTGTGACTGTAAGTGGG
GAPDH	AGGTCGGTGTGAACGGATTTG	TGTAGACCATGTAGTTGAGGTCA

**Table 2:** Effects of simvastatin on insulin content, secretion in MIN6 cells, and total insulin at low glucose levels (mean ± SD, *n* = 6, *μ*IU/mg protein).

Group	Insulin secretion	Insulin content	Total amount of insulin
Group NC	62.12 ± 4.03	540.32 ± 56.75	602.43 ± 53.41
Group A	36.82 ± 9.20	357.17 ± 34.09	393.98 ± 34.33
Group B	10.00 ± 5.90	235.53 ± 68.70	245.53 ± 63.44
Group C	4.78 ± 2.05	231.02 ± 45.13	235.80 ± 46.36
*F* value	80.16	30.26	46.04
*P* value	<0.001	<0.001	<0.001

**Table 3:** Effects of simvastatin on insulin content, secretion in MIN6 cells, and total insulin at high glucose levels (mean ± SD, *n* = 6, *μ*IU/mg protein).

Groups	Insulin secretion	Insulin content	Total amount of insulin
Group NC	98.53 ± 6.69	720.17 ± 59.66	818.69 ± 58.78
Group A	102.63 ± 23.13	471.09 ± 103.48	573.72 ± 98.43
Group B	106.49 ± 5.91	378.40 ± 75.83	484.88 ± 79.05
Group C	69.24 ± 9.25	288.29 ± 122.58	357.53 ± 114.43
*F* value	6.58	15.81	18.67
*P* value	0.007	<0.001	<0.001

**Table 4:** Effects of simvastatin on ATP levels in MIN6 cells.

Groups	ATP level (nmol/mg protein)	*N*
Group NC	73.49 ± 3.84	10
Group A	55.72 ± 4.06	10
Group B	42.79 ± 2.89	10
Group C	33.11 ± 1.75	10
*F* value	285.91	
*P* value	<0.001	
